# Case Report: Massive epistaxis from juvenile angiofibroma in an adolescent with severe haemophilia A

**DOI:** 10.12688/f1000research.20147.2

**Published:** 2019-10-04

**Authors:** Jose Florencio F. Lapeña, Olivia Agnes D. Mejia

**Affiliations:** 1 Otorhinolaryngology, Philippine General Hospital, University of the Philippines, Ermita, Manila, 1000, Philippines

**Keywords:** epistaxis, juvenile angiofibroma, haemophilia a, male adolescents, nasal endoscopy, nasal surgical procedures, computed tomography angiography

## Abstract

Epistaxis may be profuse in individuals with normal bleeding parameters, but in an individual with haemophilia, it may be life-threatening. It is even more dangerous when epistaxis is caused by an undetected concomitant juvenile angiofibroma, and only one such case has been reported in the English literature. We report another case, of an 18-year-old Filipino adolescent with severe haemophilia A who was referred for repeated massive epistaxis. The epistaxis had been attributed to his haemophilia and managed with nasal packing, multiple blood transfusions and Factor VIII administration. After two years of unsuccessful management, nasal endoscopy was performed for the first time, revealing an intranasal mass. Imaging showed a right intranasal vascular tumour supplied mainly by the right sphenopalatine artery. He subsequently underwent preoperative embolization and endoscopic excision of the tumour with Factor VIII transfused pre-, intra-, and post-operatively, and recombinant Factor VII added post-operatively. Final histopathology was consistent with juvenile angiofibroma. There has been no nasal obstruction or recurrence of epistaxis seven years since the surgery. Clinicians should be more meticulous in assessing epistaxis in any patient with a bleeding disorder and investigate more subtle symptoms such as nasal obstruction. Verification of the source by direct visualization and ancillary diagnostic techniques (such as imaging) when indicated should be the standard of care for all patients presenting with epistaxis, whether or not a concomitant bleeding disorder exists. A high index of suspicion for juvenile angiofibroma should be maintained in adolescent males with epistaxis and nasal obstruction.

## Introduction

Juvenile angiofibroma (JA) is a benign vascular tumour accounting for 0.5% of all head and neck neoplasms ^1^. It occurs almost exclusively in adolescent males nine to 19-years-old, with a mean age at diagnosis of 15 years ^2^. The clinical presentation involves unilateral epistaxis, nasal obstruction, and an intranasal mass. Epistaxis may be profuse and require nasal packing, vasopressors, antifibrinolytics and transfusions, even in individuals with normal bleeding parameters. However, with haemophilia, such epistaxis is more difficult to control and can be life-threatening. To our knowledge, only one case of JA in a haemophiliac has been reported in the English literature ^3^. We report another case here. 

## Case presentation

An 18-year-old male Filipino adolescent was referred to the Department of Otorhinolaryngology of the Philippine General Hospital for recurrent epistaxis. Previously diagnosed with severe haemophilia A at age 16, he initially presented with recurrent right nasal congestion and an episode of predominantly right-sided epistaxis described as sudden and profuse, amounting to 1500 ml. At that time, he was admitted to a provincial hospital and received blood and cryoprecipitate transfusions. Following discharge, epistaxis of 100 ml recurred almost daily, requiring nasal packs, repeated hospitalizations of one to two weeks in duration, and transfusions. Cryoprecipitate was often used to control the bleeding since plasma-derived Factor VIII (pFVIII) was seldom available due to shortage of supply and cost. His past history also included hemarthroses and gum bleeding since early childhood, but his symptoms were initially ignored and later only attributed to haemophilia although nasal congestion gradually progressed to obstruction.

After two years of such management, nasal endoscopy performed for the first time by a visiting otorhinolaryngologist revealed a right intranasal mass. He was referred to our institution and admitted with an impression of JA (Radkowski IA) and severe haemophilia A. Following admission, he suffered from hypovolemic shock several times due to difficulty in acquiring blood, cryoprecipitate and Factor VIII. With previous Factor VIII Assay levels less than 1%, 1900 units of Factor VIII concentrate were empirically administered (calculated by weight) to raise levels to normal. His condition was compounded by development of Factor VIII antibodies because of previous, repeated cryoprecipitate transfusions in a suboptimal health-care setting. Although his baseline inhibitor titre had been negative, the preoperative inhibitor titre following multiple transfusions with various blood products was positive 3.5 Bethesda units (BU), necessitating pre-, intra- and post-operative transfusion with recombinant Factor VII (rFVIIa) in addition to higher doses of Factor VIII. Unfortunately, rFVIIa only became available post-operatively.

**Figure 1.**
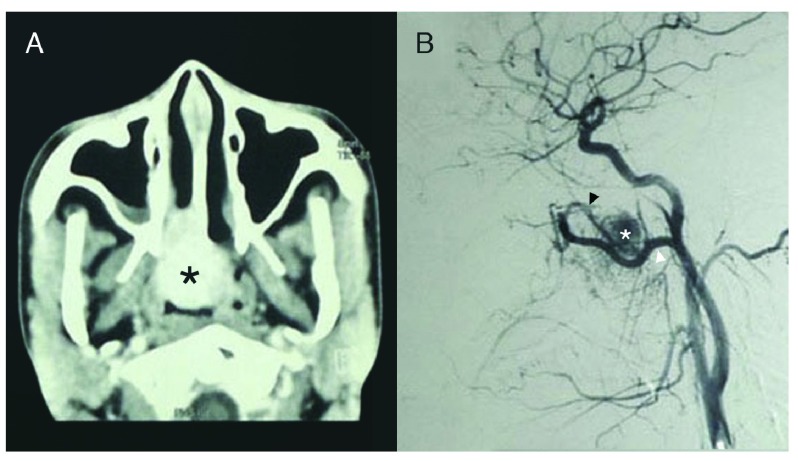
**A**. CECT Scan showing enhancing nasopharyngeal mass (asterisk) and **B**. Angiography showing vascular tumour (asterisk) supplied by sphenopalatine (black arrowhead) and internal maxillary (white arrowhead) arteries. Adobe Photoshop CC 20.01 release was used to erase identifying patient details, remove pixelated areas from black background, and enhance contrast to sharpen image (applied to entire image).

Contrast-enhanced computed tomography (CT) scans showed a hyperdense right intranasal mass corroborated by preoperative embolization angiography as an intranasal vascular tumour supplied by the right sphenopalatine artery and internal maxillary artery (IMA) ( Figure 1A and 1B). The vast majority (90%) of the blood supply arose from distal sphenopalatine branches of the right IMA, while the remaining 10% came from both ascending pharyngeal arteries ( Figure 1B).

**Figure 2.**
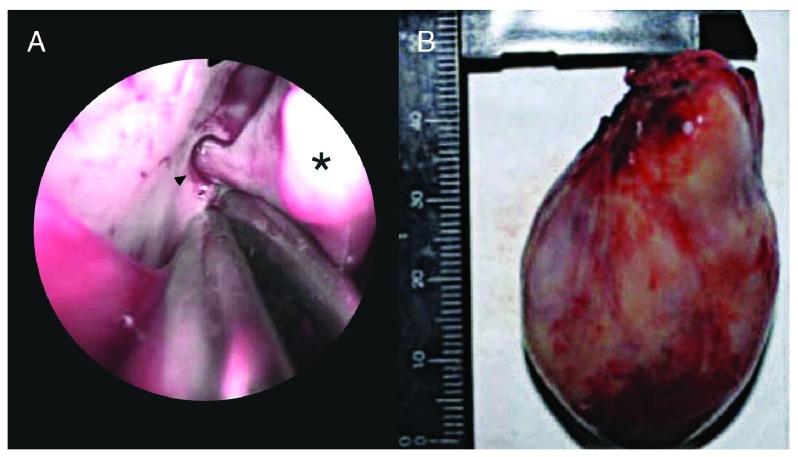
**A**. Intraoperative endoscopic view of the sphenopalatine artery (black arrowhead) supplying the mass (black asterisk) and **B**. Gross specimen measuring 4.7 × 3.2 × 2.7 cm. Adobe Photoshop CC 20.01 release was used to erase identifying patient details and sharpen the image (applied to entire image 2 **B**).

**Figure 3.**
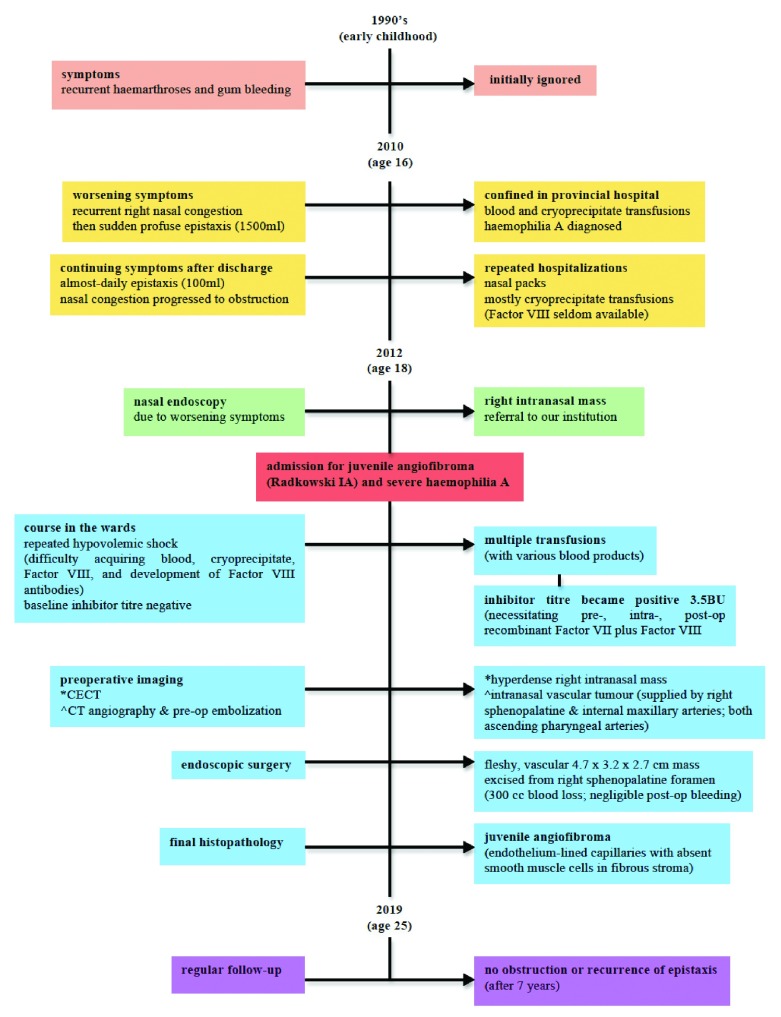
Timeline summarising important information from the case presentation.

Within 24 hours post-embolization, the patient underwent endoscopic surgery under general endotracheal anaesthesia with Sevoflurane. Factor VIII was given before, during, and after surgery, with recombinant Factor VII added post-operatively. Intraoperatively, a fleshy, vascular 4.7 × 3.2 × 2.7 cm mass was seen arising from the right sphenopalatine foramen. The sphenopalatine artery was cauterized and ligated, and the mass was delivered trans-orally ( Figure 2A and 2B). Intraoperative blood loss was 300cc and post-operative bleeding was negligible. In total, the patient received 39,500 units of commercially available pFVIII, 24 mg of rFVIIa, 22 units of packed red blood cells (PRBC), 301 units of cryoprecipitate, 1 unit of whole blood and 3 units of fresh frozen plasma (FFP). Final haematoxylin-eosin stained histopathology findings showed endothelium-lined capillaries with absent smooth muscle cells in a fibrous stroma, consistent with JA. Post-operative recovery was uneventful and the patient was discharged within a week of surgery (after two months in hospital). He has followed up regularly, with no evidence of tumour on nasal endoscopy and no recurrence of nasal obstruction or epistaxis reported by the patient for seven years. He has completed a vocational course at college and is well. Figure 3 summarizes the timeline.

## Discussion

To our knowledge, there is only one previous case of JA and concomitant haemophilia in the English literature, twice reported by Ozturk *et al.* in 1999 ^3^ and by Celiker *et al.* in 2004 ^4^. In their case, the preliminary diagnosis of JA was confirmed by biopsy at a different medical centre, where massive haemorrhage jeopardized the patient’s life. On referral to their institution, preoperative embolization, surgical excision, and adequate Factor VIII replacement saved the patient ^4^.

Similarly, significant risk to our patient’s life was posed by delayed diagnosis from hasty attribution of epistaxis to haemophilia alone, and not the possibility of a vascular tumour such as JA. Per haemophilia management guidelines, the long history of “spontaneous bleeding into joints or muscles” in our patient corresponded to the baseline Factor VIII assay clotting factor level of “<1 IU/dL or <1% of normal” seen in severe haemophilia ^5^. While recent-onset of bleeding from “mucous membranes in the mouth, gums, nose, and genitourinary tract” was serious, massive bleeding with “neck/throat” involvement was “life-threatening.” This degree of epistaxis should not have been expected in patients with haemophilia A alone, where major bleeding from these areas only occurs 5–10% of the time ^5^. Moreover, the symptom of nasal obstruction was long-overlooked. Unfortunately, two full years passed before the underlying tumour was discovered.

Current haemophilia guidelines ^5^ advise otolaryngologist referral only for “persistent or recurrent” epistaxis, but the emphasis in this recommendation is for control of bleeding only and not to investigate a different underlying cause such as JA. On the other hand, epistaxis guidelines ^6^ do recommend “anterior rhinoscopy with headlight following nasal decongestion” escalating to “rigid endoscopy or microscopy … where anterior rhinoscopy fails to identify a bleeding point.” Our experience demonstrates that vascular lesions causing epistaxis may remain undetected when presumptively attributed to pre-existing bleeding disorders and are likely to remain undetected unless sought.

In conclusion, although haemophilia guidelines do not mention vascular lesions such as JA, a high index of suspicion should be maintained in adolescent males with epistaxis and nasal obstruction. Per epistaxis guidelines, clinicians should carefully assess the cause of epistaxis in any patient with a bleeding disorder, and direct visualization of the source should be attempted (and verified by ancillary diagnostic techniques such as imaging when indicated) in all patients with epistaxis, regardless of the presence of a concomitant bleeding disorder.

## Data availability

All data underlying the results are available as part of the article and no additional data are required.

## Consent

Written informed consent for publication of his clinical details and clinical images was obtained from the patient.
