# Supplementary material for: Case Report: Massive epistaxis from juvenile angiofibroma in an adolescent with severe haemophilia A
Source: F1000Res. 2019 Oct 4;8:1593. Originally published 2019 Sep 5. [Version 2] doi: 10.12688/f1000research.20147.2 (PMC6758835; doi:10.12688/f1000research.20147.2)
Supplement: Peer review history

# Peer review: Reviewer response for version 1

**DOI:** 10.5256/f1000research.22132.r53497

**Published:** 2019-09-24

**Authors:** Robert G. Berkowitz

**Affiliations:** 1 Department of Otolaryngology, Royal Children's Hospital, Melbourne, Parkville, Vic, Australia

The authors report a case of juvenile angiofibroma (JA) occurring in a patient with haemophilia, where severe epistaxes were ascribed to the bleeding disorder and no underlying cause was sought for a period of two years.  Following transfer to the author's institution, the JA was managed successfully by surgery with pre-operative embolisation and optimisation of haemophila therapy.  There were no surgical complications and the patient has remained symptom free after long term follow-up. 

 This represents only the second reported case of JA occurring in a patient with haemophilia. The report underscores the importance of considering a separate explanation for epistaxis in a patient with an underlying coagulopathy, and particularly where there are other symptoms suggestive of intra-nasal pathology.

 The article is well written and carries a clear message. Minor points for the authors to consider providing further information are: 

- It is implied, but not actually stated, that during the two year period where the JA was overlooked, the patient's haemophilia was poorly controlled (which presumably contributed to  overlooking the diagnosis of JA). Is this the case?  
- If treatment proceeded uneventfully, why did the patient require hospitalisation for a period of 2 months?

Are enough details provided of any physical examination and diagnostic tests, treatment given and outcomes?

Yes

Is the case presented with sufficient detail to be useful for other practitioners?

Yes

Is sufficient discussion included of the importance of the findings and their relevance to future understanding of disease processes, diagnosis or treatment?

Yes

Is the background of the case’s history and progression described in sufficient detail?

Yes

Reviewer Expertise:

paediatric otolaryngology and airway disorders

I confirm that I have read this submission and believe that I have an appropriate level of expertise to confirm that it is of an acceptable scientific standard.

# Response

**Published:** 2019-10-02

**Authors:** Jose Florencio Lapeña

**Affiliations:** University of the Philippines Manila, Philippines

We thank the reviewer for his kind review and for raising minor (but important) points for consideration:

1. *"It is implied, but not actually stated, that during the two year period where the JA was overlooked, the patient's haemophilia was poorly controlled (which presumably contributed to overlooking the diagnosis of JA). Is this the case?"*

Yes, " *during the two year period where the JA was overlooked, the patient's haemophilia was poorly controlled (which presumably contributed to  overlooking the diagnosis of JA)*." We were hoping the first paragraph conveyed a sense of this poor control without being overly dramatic. As mentioned in the Case presentation, this can be attributed to inadequate factor VIII replacement therapy (" *seldom available due to shortage of supply and cost*").  Indeed, " *this makes the case even more interesting, and highlights that not only when there is a known coagulopathy present, and even when it is not being adequately treated, a possible intra-nasal cause should still be considered.*" 

2. *"If treatment proceeded uneventfully, why did the patient require hospitalisation for a period of 2 months?"*

I hope we did not give the impression that " *treatment proceeded uneventfully*." Our " *patient require(d) hospitalisation for a period of 2 months*" because of a very stormy preoperative course wherein " *he suffered from hypovolemic shock several times due to difficulty in acquiring blood*" and blood products, and " *his condition was compounded by development of Factor VIII antibodies.*" He also " *received 39,500 units of commercially available pFVIII, 24 mg of rFVIIa, 22 units of packed red blood cells (PRBC), 301 units of cryoprecipitate, 1 unit of whole blood and 3 units of fresh frozen plasma (FFP)*."  

We have revised the Case presentation by adding the statement " *Post-operative recovery was uneventful and the patient was discharged within a week of surgery (after two months in hospital)*." to clarify that the prolonged hospitalisation was due to the stormy pre-operative course.

# Peer review: Reviewer response for version 1

**DOI:** 10.5256/f1000research.22132.r53498

**Published:** 2019-09-23

**Authors:** J. Paul Moxham

**Affiliations:** 1 Division of Otolaryngology-Head and Neck Surgery, University of British Columbia, Vancouver, BC, Canada

This is an excellent and well written case report about a young adult with a hematologic disorder and a coexisting angiofibroma. It delves into the difficulties this case presents to the treating surgeon, reviews the relevant literature (of which there is only one previous report), and reminds us that just because someone has a bleeding disorder does not mean they cannot also have a rare vascular tumour.

Are enough details provided of any physical examination and diagnostic tests, treatment given and outcomes?

Yes

Is the case presented with sufficient detail to be useful for other practitioners?

Yes

Is sufficient discussion included of the importance of the findings and their relevance to future understanding of disease processes, diagnosis or treatment?

Yes

Is the background of the case’s history and progression described in sufficient detail?

Yes

Reviewer Expertise:

I am an Academic Pediatric Otolaryngologist at BC Children's Hospital and an Associate Clinical Professor of Surgery at the University of British Columbia in Vancouver, Canada. I am a member of the Triological Society and the American Society of Pediatric Otolaryngology. My main areas of interest are bone grow factors in craniofacial models.

I confirm that I have read this submission and believe that I have an appropriate level of expertise to confirm that it is of an acceptable scientific standard.

# Response

**Published:** 2019-10-02

**Authors:** Jose Florencio Lapeña

**Affiliations:** University of the Philippines Manila, Philippines

We thank the reviewer for his clear and concise review, recapitulating the main theme and "take-home" message of our case report " *that just because someone has a bleeding disorder does not mean they cannot also have a rare vascular tumour*."

# Peer review: Reviewer response for version 1

**DOI:** 10.5256/f1000research.22132.r53496

**Published:** 2019-09-09

**Authors:** Alberto Maria Saibene

**Affiliations:** 1 Department of Otolaryngology, San Paolo Hospital, University of Milan, Milan, Italy

The Authors present a compelling case report where the concomitance of a rare sinonasal vascular tumour, i.e. a juvenile angiofibroma, and haemophilia A delayed diagnosis and complicated clinical management. As the authors correctly demonstrate in the report, a sub-optimal healthcare setting led to haemophilia first diagnosis delay and, foremost, delayed the identification of the neoplasm despite the recurrence of profuse bleeding.

 The article is well written, both by a grammar and literary standpoint, and present all clinical information on diagnosis and management in a complete fashion.

 The clinical management is sound and clinical decisions are consistent with current guidelines and good clinical practice.

 The article could be publishable in this present form, but I'd like to point out a couple of ideas that might add some teaching relevance to the article:

- First of all, it might be worth mentioning that in good rhinologic practice, performing sinonasal tumors biopsies without adequate imaging can result to harmful or fatal incidents. Had the clinicians decided to perform a biopsy in an outpatient setting, a massive epistaxis could have led to serious complications for this patient, without helping further clinical decisions. This is the case of the - correctly cited - case report already published by Ozturk.
- Secondly, while it is true that haemophilia guidelines do not advise routine evaluation for epistaxis, on the other hand epistaxis guidelines ^1^ do recommend for a thorough evaluation of the patient in order to identify the bleeding source in all case, starting with anterior rhinoscopy and escalating to nasal endoscopy whenever the source of bleeding cannot be easily identified. Therefore, it is worth mentioning that correct management of all epistaxis cases required identification of the bleeding source.
- Last, while the CT scan the authors provided allows a good depiction of the clinical picture, it would be interesting to see whether the CT scans showed enlargement of the sphenopalatine foramen. While such enlargement is not constant in all JA patients, an enlargement >3mm in presence of a unilateral sinonasal mass can point the diagnosis towards JA.

In conclusion, this is an extremely interesting article well deserving publication, with an interesting teaching value that could be further increased with some more information as above stated.

Are enough details provided of any physical examination and diagnostic tests, treatment given and outcomes?

Yes

Is the case presented with sufficient detail to be useful for other practitioners?

Yes

Is sufficient discussion included of the importance of the findings and their relevance to future understanding of disease processes, diagnosis or treatment?

Yes

Is the background of the case’s history and progression described in sufficient detail?

Yes

Reviewer Expertise:

Otolaryngology, rhinology, head and neck surgery

I confirm that I have read this submission and believe that I have an appropriate level of expertise to confirm that it is of an acceptable scientific standard.

## References

[1] : The British Rhinological Society multidisciplinary consensus recommendations on the hospital management of epistaxis. *J Laryngol Otol* .131(12) : 10.1017/S0022215117002018 1142-1156 10.1017/S0022215117002018 29280691

# Response

**Published:** 2019-10-02

**Authors:** Jose Florencio Lapeña

**Affiliations:** University of the Philippines Manila, Philippines

We thank the reviewer for his excellent review and valuable comments and recommendations: 

1. " *First of all, it might be worth mentioning that in good rhinologic practice, performing sinonasal tumors biopsies without adequate imaging can result to harmful or fatal incidents. Had the clinicians decided to perform a biopsy in an outpatient setting, a massive epistaxis could have led to serious complications for this patient, without helping further clinical decisions. This is the case of the - correctly cited - case report already published by Ozturk.*"

Yes, indeed. A biopsy in the case of our patient would have been catastrophic (and we have witnessed this happen to patients of other unsuspecting physicians). Any nasal mass suspicious for angiofibroma should not be manipulated (unless in the operating theatre under double set-up). However, we felt that mentioning this important management caveat would detract from the main message of initial diagnosis necessitating visualising the bleeding source (directed toward general practitioners, primary care providers, family physicians, and paediatricians), and opted to reiterate the point in our response to the review instead.

2.  " *Secondly, while it is true that haemophilia guidelines do not advise routine evaluation for epistaxis, on the other hand epistaxis guidelines (1) do recommend for a thorough evaluation of the patient in order to identify the bleeding source in all case, starting with anterior rhinoscopy and escalating to nasal endoscopy whenever the source of bleeding cannot be easily identified. Therefore, it is worth mentioning that correct management of all epistaxis cases required identification of the bleeding source*."

We have added the statement " *On the other hand, epistaxis guidelines(6) do recommend 'anterior rhinoscopy with headlight following nasal decongestion' escalating to 'rigid endoscopy or microscopy … where anterior rhinoscopy fails to identify a bleeding point.' *" to the Discussion, for which the additional reference (6) provided by the reviewer was cited:

National ENT Trainee Research Network. The British Rhinological Society multidisciplinary consensus recommendations on the hospital management of epistaxis. J Laryngol Otol. 2017;131(12):1142-1156. 10.1017/S0022215117002018.

3.  " *Last, while the CT scan the authors provided allows a good depiction of the clinical picture, it would be interesting to see whether the CT scans showed enlargement of the sphenopalatine foramen. While such enlargement is not constant in all JA patients, an enlargement >3mm in presence of a unilateral sinonasal mass can point the diagnosis towards JA."*

We totally agree that the educational value of the article can be enhanced by mentioning that " *in the presence of a unilateral sinonasal mass*," such an enlargement of the sphenopalatine foramen " *can point to the diagnosis of JA*." However (and in relation to the first point above), we opted to maintain the primary focus of our discussion and perhaps encourage our non-otolaryngologist colleagues to consider urgent referrals to ENT surgeons following initial diagnosis in such cases.
